# Patterns of HIV-1 Viral Load Suppression and Drug Resistance During the Dolutegravir Transition: A Population-based Longitudinal Study

**DOI:** 10.1093/cid/ciag161

**Published:** 2026-03-13

**Authors:** Michael A Martin, Alexandra Blenkinsop, Michelle Moffa, Steven James Reynolds, Fred Nalugoda, Thomas C Quinn, Godfrey Kigozi, Robert Ssekubugu, Ravindra K Gupta, Nicholas E Grayson, George MacIntyre-Cockett, Joseph Kagaayi, Gertrude Nakigozi, Lucie Abeler-Dörner, Christophe Fraser, Oliver Ratmann, Aaron A R Tobian, Oliver Laeyendecker, Sikhulile Moyo, Caitlin E Kennedy, David Bonsall, Ronald Moses Galiwango, M Kate Grabowski

**Affiliations:** Department of Pathology, Johns Hopkins School of Medicine, Baltimore, Maryland, USA; Department of Epidemiology, Johns Hopkins Bloomberg School of Public Health, Baltimore, Maryland, USA; Department of Mathematics, Imperial College London, London, United Kingdom; Imperial-X, Imperial College London, London, United Kingdom; Johns Hopkins School of Medicine, Baltimore, Maryland, USA; Rakai Health Sciences Program, Kalisizo, Uganda; Division of Infectious Disease, Department of Medicine, Johns Hopkins School of Medicine, Baltimore, Maryland, USA; Division of Intramural Research, National Institute of Allergy and Infectious Diseases, National Institutes of Health, Bethesda, Maryland, USA; Rakai Health Sciences Program, Kalisizo, Uganda; Rakai Health Sciences Program, Kalisizo, Uganda; Division of Infectious Disease, Department of Medicine, Johns Hopkins School of Medicine, Baltimore, Maryland, USA; Division of Intramural Research, National Institute of Allergy and Infectious Diseases, National Institutes of Health, Bethesda, Maryland, USA; Rakai Health Sciences Program, Kalisizo, Uganda; Rakai Health Sciences Program, Kalisizo, Uganda; Department of Medicine, University of Cambridge, Cambridge, United Kingdom; Africa Health Research Institute, KwaZulu-Natal, South Africa; Centre for Human Genetics, Nuffield Department of Medicine, University of Oxford, Oxford, United Kingdom; Centre for Human Genetics, Nuffield Department of Medicine, University of Oxford, Oxford, United Kingdom; Makerere University School of Public Health, Kampala, Uganda; Rakai Health Sciences Program, Kalisizo, Uganda; Pandemic Sciences Institute, Nuffield Department of Medicine, University of Oxford, Oxford, United Kingdom; Pandemic Sciences Institute, Nuffield Department of Medicine, University of Oxford, Oxford, United Kingdom; Department of Mathematics, Imperial College London, London, United Kingdom; Imperial-X, Imperial College London, London, United Kingdom; Department of Pathology, Johns Hopkins School of Medicine, Baltimore, Maryland, USA; Division of Infectious Disease, Department of Medicine, Johns Hopkins School of Medicine, Baltimore, Maryland, USA; Division of Intramural Research, National Institute of Allergy and Infectious Diseases, National Institutes of Health, Bethesda, Maryland, USA; Botswana Harvard Health Partnership, Gaborone, Botswana; Department of Immunology and Infectious Diseases, Harvard T. H. Chan School of Public Health, Boston, Massachusetts, USA; Department of Pathology, Division of Medical Virology, Faculty of Medicine and Health Sciences, Stellenbosch University, Cape Town, South Africa; School of Health Systems and Public Health, University of Pretoria, Pretoria, South Africa; Department of International Health, Johns Hopkins Bloomberg School of Public Health, Baltimore, Maryland, USA; Centre for Human Genetics, Nuffield Department of Medicine, University of Oxford, Oxford, United Kingdom; Rakai Health Sciences Program, Kalisizo, Uganda; Department of Pathology, Johns Hopkins School of Medicine, Baltimore, Maryland, USA; Department of Epidemiology, Johns Hopkins Bloomberg School of Public Health, Baltimore, Maryland, USA; Rakai Health Sciences Program, Kalisizo, Uganda

**Keywords:** HIV, drug resistance, viral suppression, population-based, Uganda

## Abstract

**Background:**

Data on the population-scale impact of dolutegravir (DTG)-based HIV regimens in sub-Saharan Africa are extremely limited. We used data from a surveillance cohort in southern Uganda to assess viral suppression and antiretroviral (ART) resistance over 10-years alongside DTG scale-up.

**Methods:**

Consenting participants in the population-based Rakai Community Cohort Study between August 2011 and March 2023 aged 15–49 completed questionnaires and provided samples for HIV testing, viral load quantification, and viral deep-sequencing. We collected data on DTG utilization at HIV care clinics. We estimated the prevalence of HIV suppression and ART resistance using robust Poisson regression. Bayesian logistic regression quantified associations between resistance and individual-level suppression across surveys.

**Results:**

Among 8781 people with HIV (PWH), suppression increased from 57.1% (2014, 95% confidence interval [CI], 55.4%–58.8%) to 90.3% (2022, 95% CI, 89.2%–91.4%). By 2020 84.4% (95% CI, 83.7%–85.2%) and 64.6% (95% CI, 63.9%–65.3%) of men and women on ART were on DTG. Among treatment-experienced viremic PWH, any intermediate/high resistance decreased from 51.1% (95% CI, 40.7%–64.2%, 2014) to 27.9% (95% CI, 21.3%–36.5%, 2022). Two of 258 (0.8%) 2022 participants harbored intermediate/high-level DTG resistance (inQ148R, inE138K, and inG140A). inS153Y (2-fold INSTI resistance) was observed in 23/306 (7.5%) of viremic individuals, with evidence of transmission. By 2022, NNRTI/NRTI resistance was not associated with a reduction in individual-level suppression (risk ratios: 1.15, 95% HPD: 0.93–1.39; 1.14, 0.86–1.42).

**Conclusions:**

Viral suppression increased during the DTG transition with minimal emerging intermediate/high-level resistance. Falling resistance among treatment-experienced PWH underscores the role of ART adherence in reducing viremia. The emergence of inS153Y justifies continued surveillance.

## Teaser

Resistance-attributed treatment failure during scale-up of DTG-based HIV treatment is rare in southern Uganda. Most people with viremic HIV are susceptible to first-line treatment. Rapid emergence and transmission of low-level DTG resistance is a cautionary signal for high-burden settings.

## Graphical Abstract

**Graphical Abstract:**
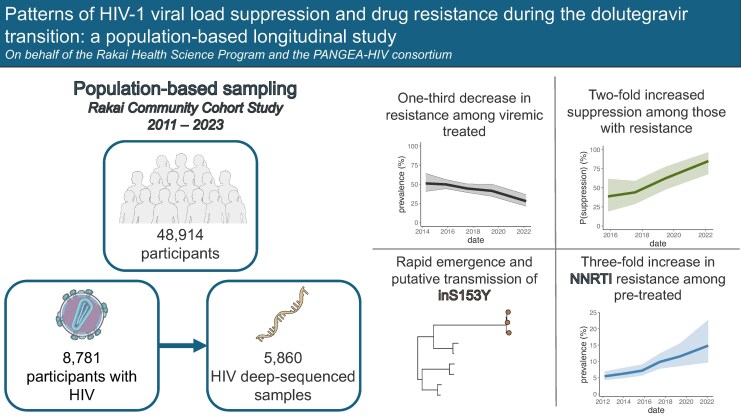

Antiretroviral therapy (ART) suppresses human immunodeficiency virus-1 (HIV-1) viral load, slowing disease progression [1] and preventing transmission [2]. Pre-2018 the World Health Organization (WHO) recommended efavirenz (EFV, a non-nucleoside reverse transcriptase inhibitor, NNRTI) with 2 nucleoside reverse transcriptase inhibitors (NRTIs) as first-line HIV treatment. Due to increased pre-treatment NNRTI resistance [3], recommended first-line regimens transitioned to the integrase strand transfer inhibitor (INSTI) dolutegravir (DTG) with NRTIs in 2018.

Studies of care-seeking individuals have indicated increased viral suppression among adults with HIV initiating or transitioning to DTG across sub-Saharan Africa [4–8]. In contrast, multi-national WHO data do not report significant trends toward increased suppression among those on DTG through 2022 [9]. While emergent DTG resistance has been observed in individuals with virological failure [10–13] there is considerable uncertainty in the true prevalence as clinic-based studies do not capture people unengaged or transiently engaged in therapy and therefore have incomplete denominators. Further, real-world data on the prevalence of pre-treatment NNRTI resistance following the DTG transition is limited, despite implications for the efficacy of long-acting injectable cabotegravir (CAB) in combination with rilpivirine, an NNRTI.

Population-based studies that sample individuals regardless of HIV sero- or treatment-status can quantify the impact of DTG on population-level virological outcomes, irrespective of treatment-seeking behavior. We used epidemiological and virological data collected from adults with HIV who participated in a population-based open-cohort study in southern Uganda to assess population- and individual-level viral suppression and ART resistance patterns between 2011 and 2023, concurrent with DTG scale-up. Previous work in this cohort preceding the DTG transition has shown a 2-fold increase in NNRTI resistance among pre-treatment people with HIV (PWH) during the expansion of treatment and prevention programs [14]. Here, we utilized additional data collected during the 3-year scale-up of DTG in this setting (2019–2022) providing insights into the real-world adoption of DTG regimens, their efficacy in moving Uganda closer to UNAIDS 95-95-95 goals, and implications for future therapy policies in sub-Saharan Africa.

## METHODS

### Study Design and Participants

The RCCS is a population-based open-cohort study conducted every 18–24 months in inland (HIV prevalence ∼14%–17%) and Lake Victoria fishing (∼42%) communities in southern Uganda [15]. During surveys households in participating communities were censused and consenting residents aged 15–49 were invited to complete a structured sociodemographic, behavioral, and health questionnaire that includes questions on current and past ART use. Participants provided samples for HIV testing, viral load quantification, and viral deep-sequencing. Participants were considered pre-treatment at a given survey if they were seropositive at that round and reported never having been on ART and had a viral load >1000 copies/mL at that and all previous rounds in which they participated. The analysis period included the 5 most recent surveys (10 August 2011 through 7 March 2023); however, we used all surveys (beginning 5 November 1994) to categorize treatment exposure. We refer to surveys by the year of the median interview date (Supplementary Table 1; 2012, 2014, 2015, 2017, 2019, and 2022).

Viremia was defined as a viral load >1000 copies/mL. As viral loads in the 2012 survey were not routinely measured for all participants, we imputed missing 2012 viremia status among pre-treatment individuals (n = 792/2,015, Supplementary Methods). Observations with missing viral load data (97/16 885) in later rounds were dropped from analyses of viremia status.

Because the RCCS does not collect data on individual drug regimens, we evaluated trends in DTG use among attendees from the 20 most commonly accessed HIV care clinics among RCCS participants. Among clinics providing data, missing data on type of regimen use were minimal (1/161 of all quarter-years stratified by sex) and were dropped.

### HIV Deep-sequence Based Drug-resistance Prediction

HIV deep-sequencing from venous blood samples was performed through the Phylogenetics and Networks for Generalized HIV Epidemics in Africa consortium (PANGEA-HIV) using an overlapping amplicon [16] or bait-capture (veSEQ-HIV [17]) approach. Non-RCCS HIV sequence data obtained from individuals residing in surrounding areas through complementary RHSP studies between 2012 and 2023 was also incorporated into genetic and phylogenetic clustering analyses. Complete polymerase sequences were used to calculate pairwise genetic distances and infer phylogenies (Supplementary Methods).

Drug-resistance mutations (DRMs) were identified using drmSEQ (Supplementary Methods) with a ≥10 and ≥5% of PCR-deduplicated reads threshold [18]. Mutations were scored according to the Stanford University HIV Drug Resistance Database v.9.6 (Supplementary Tables 2 and 3) [19, 20]. We considered a score sum of ≥30 as (intermediate/high-level) resistant and between 10 and 29 as low-level resistant.

### Study Outcomes and Statistical Methods

We estimated the prevalence of HIV, treatment-experienced HIV, and suppressed HIV among all study participants with survey round as a categorical predictor. We then estimated the prevalence of ever having been on treatment and of viremia among PWH and the prevalence of viremia among treatment-experienced PWH (txPWH).

The primary outcomes of this study were overall and class-specific prevalence of intermediate/high-level INSTI, NNRTI, NRTI, and PI resistance among viremic txPWH and among pre-treatment PWH (ptPWH). To account for missing resistance prediction among some viremic PWH, we employed inverse probability weighting (Supplementary Methods). We also evaluated whether overall resistance among txPWH and NNRTI resistance among ptPWH was associated with age, community type, sex, and viral subtype in both univariate and bivariate (with survey) models. Among viremic txPWH, we further estimated the prevalence of multi- and single-class resistance. Finally, we estimated the prevalence of individual DRMs among viremic txPWH and ptPWH using participant-visits with successful resistance prediction to all drug classes. We restricted analyses of viremic txPWH to 2014 and later due to missing 2012 viral loads.

All prevalence estimates were generated using robust Poisson regression with a log-link [21]. Generalized estimated equations were used as individuals can participate in multiple surveys, selecting correlation structure through quasi-likelihood information criterion. Independence was assumed for rare outcomes (<20 events) to ensure convergence. We also used an independence correlation structure when estimating DRM prevalence to ensure convergence, particularly for rare mutations.

We quantified the individual-level probability of suppression among viremic PWH between successive survey rounds depending on survey round and resistance to NNRTI or NRTI-based regimens in a mixed-effects Bayesian logistic regression. We accounted for missing data due to drop-outs between surveys and missing viral genomic-based resistance prediction using post-stratification weighting by survey, age category, community type, and sex (Supplementary Methods).

## RESULTS

A total of 48 914 people, including 8781 (17.95%) with HIV contributed 109 328 participant-visits between 15 August 2011 and 23 February 2023 (Supplementary Figures 1–7). Population demographics remained stable (Supplementary Table 4), however, the median (IQR) age of participants with HIV increased from 32 (10) to 37 (11) years (Supplementary Table 5).

**Figure 1.**
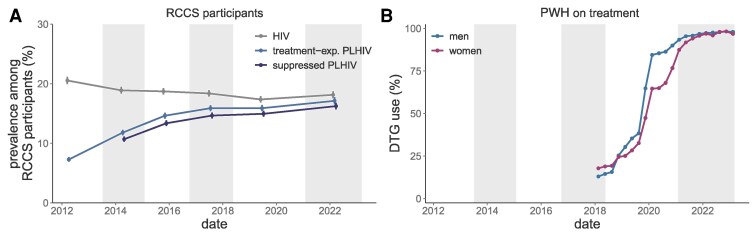
ART scale-up in the Rakai Community Cohort Study (RCCS) 2012-2022. *A*, Temporal trends in the prevalence of HIV (top), prevalence of PWH who ever report being on ART (middle), and prevalence of virally suppressed HIV (bottom). Prevalence estimates were generated using Poisson regression with robust standard errors with survey as a predictor variable. Generalized estimating equations with correlation structure selection by quasi information criterion value (independence) were used to account for repeat participants across study rounds. *B*, Proportion of PWH on DTG-based regimens at the top 14 clinics serving RCCS participants stratified by sex. Vertical error bars indicate the Wald 95% confidence interval for the mean value but for some estimates do not extend past the size of the point. Shading corresponds to the range of interview dates for alternating RCCS surveys.

The prevalence of HIV among participants decreased slightly from 20.5% in 2012 (95% confidence interval [CI], 19.9%–21.2%) to 18.1% in 2022 (95% CI, 17.6%–18.7%, Figure 1*A* and Supplementary Table 6), while the prevalence of ever having been on ART among PWH increased from 35.4% (95% CI, 33.9%–37.1%) to 94.4% (95% CI, 93.6%–95.2%, Supplementary Table 7). Consequently, suppression among all PWH increased from 57.1% (95% CI, 55.4%–58.8%) to 90.3% (95% CI, 89.2%–91.4%) between 2014 and 2022. HIV viremia among all study participants, which informs the risk of transmission, decreased 4.6-fold (95% CI, 4.06–5.21) reaching 1.7% (95% CI, 1.6%–2.0%) by 2022. Due to ART scale-up, a history of treatment among those with viremia increased 3.14-fold (prevalence ratio [PR] 95% CI, 2.6–3.80), from 13.4% (95% CI, 11.7%–15.3%) in 2014 to 42.1% (95% CI, 36.7%–48.3%) by 2022. Among txPWH, suppression reached 95.4% (95% CI, 94.6%–96.1%) by 2022, an increase from 93.6% (95% CI, 92.8%–94.4%) in 2019, driven by an increase in viral loads ≤200 copies/mL (Supplementary Table 8).

Overall, 14 clinics serving 74.1% of 2022 RCCS participants on treatment provided DTG prescription data (Figure 1*B* and Supplementary Table 9). DTG use among PWH on treatment was 53.6% (13 248/24 735) by the end of 2019 and 97.2% (26 831/27 597) by mid-2022 (Supplementary Table 10). Adoption was slower among women (adjusted *P* value = .0002) but reached parity by 2022. DTG adoption was generally consistent across surveyed facilities (Supplementary Figure 8).

Deep-sequence based drug-resistance prediction was attempted on 936/963 (97.2%) of participant-visits contributed by viremic txPWH and 4943/4998 (98.9%) contributed by ptPWH (Supplementary Tables 11 and 12). Coverage was sufficient to predict resistance to ≥1 drug class for 783 txPWH participant-visits (83.7%) and for 3920 ptPWH (79.3%). Sequencing success was better at higher viral loads as compared with lower with veSEQ-HIV as compared with the amplicon-based protocol, and for INSTIs compared with other classes. Among the 348 (37.2%) sequenced participant-visits contributed by individuals who initiated treatment in the cohort, median (IQR) time on treatment was 0.90 (0.20) years.

**Figure 2.**
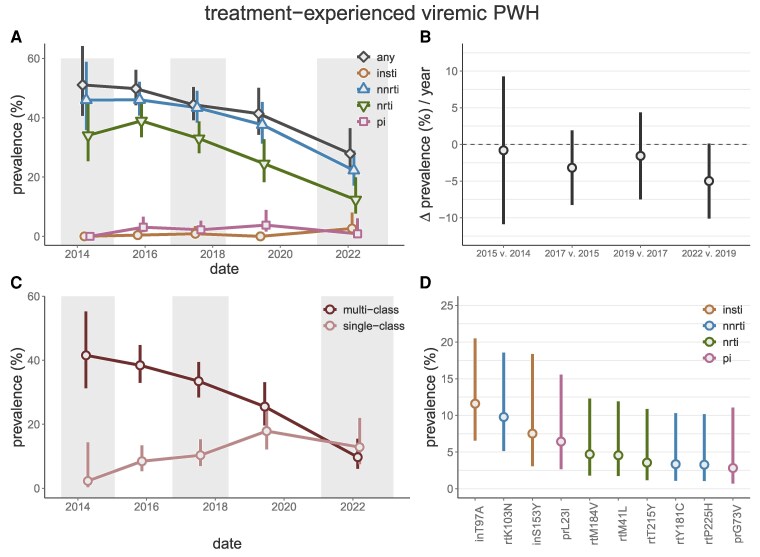
ART resistance among treatment-experienced viremic PWH in the Rakai Community Cohort Study. *A*, Prevalence of resistance to any drug class (diamonds), integrase strand transfer inhibitors (INSTIs, circles), non-nucleoside reverse transcriptase inhibitors (NNRTIs, upright triangles), nucleoside reverse transcriptase inhibitors (NRTIs, inverted triangles), and protease inhibitors (PIs, squares) by survey among treatment-experienced viremic PWH. *B*, Difference between the estimated prevalence of resistance to any drug class in consecutive surveys, adjusted for the time between surveys. *C*, Prevalence of multiclass and single-class resistance among treatment-experienced viremic PWH. Multiclass refers to any combination of simultaneous resistance to any of the 4 drug classes. *D*, Prevalence of resistance mutations among treatment-experienced viremic PWH, colored by the class of drug to which a given mutation confers resistance. Vertical bars extend to the Wald 95% confidence intervals. *A* and *C*, Generalized estimating equations with correlation structure selection by quasi information criterion value (Any, NRTI, NNRTI: AR1) or independence for outcome measures with <20 events and for all mutations (*D*) were used to account for repeat participants across study rounds. Shading corresponds to the range of interview dates for alternating RCCS surveys.

Between 2014 and 2022, the prevalence of resistance to any ARTs among viremic txPWH decreased from 51.1% (95% CI, 40.7%–64.2%) to 27.9% (95% CI, 21.3%–36.5%, Figure 2*A* and Supplementary Table 13). The largest round-over-round drop (4.99% year^−1^, 95% CI, −10.10%–0.11% year^−1^) happened between 2019 and 2022 (*P* value = .0325; Figure 2*B*). Consequently, in 2022, the majority of viremic txPWH lacked detectable resistance, which was robust to the choice of variant calling threshold (Supplementary Figure 9). Resistance among txPWH did not vary by age or viral subtype but was slightly more common among participants in inland, as compared with fishing, communities (Supplementary Table 14; adjusted *P* value = .053) and women (adjusted *P* value = .0001). The vast majority of observed txPWH resistance was NNRTI (2022 prevalence 22.2%, 95% CI, 17.1%–28.9%) and NRTI (12.3%, 95% CI, 7.7%–19.9%). While multiclass NNRTI/NRTI resistance predominated in 2012 (Figure 2*C* and Supplementary Table 15), it declined precipitously during the study such that in 2022 single-class resistance was slightly more common. INSTI and PI resistance remained <4%, with only 2/108 (1.85%) of 2022 txPWH exhibiting intermediate/high-level DTG resistance (Table 1).

**Table 1.** Demographic Data on Participants With Low-, Intermediate-, or High-level Dolutegravir (DTG) Resistance

ID	Sex	Age	Round	INSTI Mutations	Other Mutations	Treatment Status	First ARV	Last HIV−	First HIV+
16284	F	(24,34]	2012	inQ148R	…	Pre-treatment	…	…	1999
13513	F	(14,24]	2015	inR263K	prK43T	Treatment-experienced	2013	2011	2013
19515	F	(24,34]	2022	inS153Y	…	Pre-treatment	…	2014	2021
19372	F	(14,24]	2022	inS153Y	prL23I	Pre-treatment	…	…	2022
21793	M	(34,49]	2022	inS153Y	prG73 V, prL23I	Pre-treatment	…	2019	2022
40934	M	(24,34]	2022	inS153Y	…	Pre-treatment	…	…	2021
16208	M	(14,24]	2022	inS153Y	rtF227L, rtK103N	Pre-treatment	…	2017	2021
36454	F	(24,34]	2022	inS153Y	…	Pre-treatment	…	2018	2021
42998	F	(14,24]	2022	inS153Y	…	Pre-treatment	…	…	2022
31609	F	(34,49]	2022	inS153Y	…	Pre-treatment	…	2018	2021
30284	F	(24,34]	2022	inS153Y	…	Pre-treatment	…	2017	2021
39941	F	(24,34]	2022	inS153Y	…	Pre-treatment	…	…	2021
47286	M	(34,49]	2022	inS153Y	rtF77L	Treatment-experienced	2017	…	2017
37114	F	(14,24]	2022	inS153Y	rtK103N	Treatment-experienced	2021	…	2021
04449	F	(14,24]	2022	inE138 K, inQ148 K, inG140A	rtM41L, rtG190S, rtM184V	Treatment-experienced	2020	…	2020
36345	M	(14,24]	2022	inS153Y	…	Treatment-experienced	2021	…	2021
15335	M	(24,34]	2022	inS153Y	…	Treatment-experienced	2019	2011	2015
35550	F	(24,34]	2022	inS153Y	…	Treatment-experienced	2018	…	2014
41004	F	(14,24]	2022	inT97A, inS153Y	rtV179L	Treatment-experienced	2021	…	2021
08593	F	(34,49]	2022	inQ148R, inE138K	rtM184V	Treatment-experienced	2014	2006	2008
16868	M	(34,49]	2022	inS153Y	…	Treatment-experienced	2019	…	2015

Among viremic txPWH in 2022, the most common resistance mutation was the polymorphic accessory mutation inT97A (11.6%, 95% CI, 6.6%–20.5%), which confers low-level INSTI (eg elvitegravir [22]) resistance and has been associated with DTG failure in combination with other DRMs (Figure 2*D* and Supplementary Tables 16 and 17) [11, 19, 20]. The 2-fold DTG accessory resistance mutation inS153Y [23] was also observed in 7/84 (8.3%)) of viremic txPWH in 2022, despite not being observed previously (2022 prevalence: 7.5%, 95% CI, 3.1%–18.4%) or elsewhere [24]. Both 2022 treatment-experienced participants with intermediate/high-level DTG resistance had inE138K, one with inQ148R and one with inG140A and inQ148R (Table 1) [25]. Both individuals had multiclass NNRTI/NRTI resistance in surveys pre-dating the DTG scale-up (Supplementary Table 18).

The majority of NNRTI and NRTI resistance in this population was attributable to rtK103N (9.8%, 95% CI, 5.2%–18.6%, high-level EFV and nevirapine resistance [26]), rtM184V (4.7%, 95% CI, 1.8%–12.3%], high-level 3TC and emtricitabine resistance [27]), and rtM41L (4.6% [95% CI, 1.7%–11.9%]), a thymidine analog mutation [28]).

**Figure 3.**
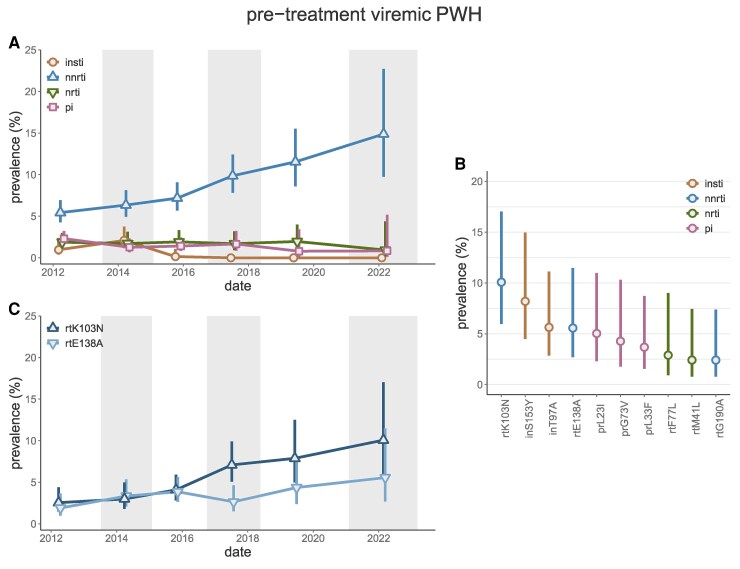
ART resistance among pre-treatment viremic PWH in the Rakai Community Cohort Study. *A*, Prevalence of resistance to integrase strand transfer inhibitors (INSTIs, circles), non-nucleoside reverse transcriptase inhibitors (NNRTIs, upright triangles), nucleoside reverse transcriptase inhibitors (NRTIs, inverted triangles), and protease inhibitors (PIs, squares) by survey among treatment-experienced viremic PWH. Generalized estimating equations with correlation structure selection by Quasi Information Criterion value (NRTI, PI: AR1, NRTI: exchangeable) or independence for outcome measures with <20 events. *B*, Prevalence of resistance mutations among pre-treatment viremic PWH, colored by the class of drug to which a given mutation confers resistance. *C*, Prevalence of rtK103N and rtE138A mutations by survey among pre-treatment viremic PWH. Bars extend to the Wald 95% confidence intervals. Bars extend to the Wald 95% confidence intervals. Shading corresponds to the range of interview dates for alternating RCCS surveys.

Despite the transition to DTG-based first-line regimens, NNRTI resistance among ptPWH increased 2.74-times (95% CI, 1.68–4.45) throughout the study period (Figure 3*A* and Supplementary Table 19). Between 2019 and 2022 alone pre-treatment NNRTI resistance increased from 11.5% (95% CI, 8.6%–15.5%) to 14.9% (95% CI, 9.7%–22.7%). NNRTI resistance was slightly more common among women (adjusted *P* value = .022; Supplementary Table 20). In contrast, intermediate/high-level pre-treatment NRTI/INSTI/PI resistance remained consistently below 2.5%. We did not observe intermediate/high-level DTG resistance among ptPWH.

NNRTI resistance in this group of individuals was predominantly attributed to rtK103N (2022 prevalence: 10.1%, 95% CI, 6%–17%, Figure 3*B* and 3*C* and Supplementary Tables 21 and 22) and rtE138A (5.6%, 95% CI, 2.7%–11.5%). The increase in NNRTI resistance was driven by rtK103N which increased 3.95-fold (95% CI, 1.86–8.4) between 2012 and 2022 whereas rtE138A increased only modestly (PR: 2.92, 95% CI, 1.1–7.73). While not solely conferring intermediate/high-level INSTI resistance, we observed inT97A and inS153Y in 10 and 8 of 133 (7.5% and 6%) pre-treatment PWH in 2022 (2022 prevalence: 8.2%, 95% CI, 4.5%–15% and 5.6%, 95% CI, 2.9%–11.1%). While inT97A remained stable over the study period (PR vs. 2012: 0.69, 95% CI, 0.33–1.45), inS153Y was not observed prior to 2022, consistent with observations made among viremic txPWH (Table 1).

**Figure 4.**
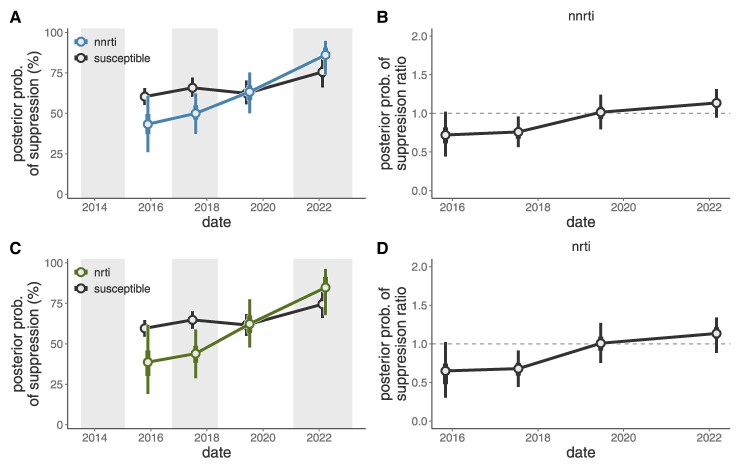
Probability of viral load suppression among Rakai Community Cohort Study participants who were viremic in the proceeding survey. *A*, Probability of viral load suppression among PWH who participated in the RCCS and were viremic in the preceding survey, stratified by NNRTI resistance (blue) or susceptibility (black). *B*, Posterior ratio of the probability of suppression among those with NNRTI resistance compared with those without in each survey. *C*, Probability of viral load suppression among PWH who participated in the RCCS and were viremic in the preceding survey, stratified by NRTI resistance (green) or susceptibility (black). *D*, Risk ratio of the probability of suppression among those with NRTI resistance compared with those without in each survey. Median value of the posterior distribution plotted as the central estimate and bars extend to the 50% and 95% highest posterior density. Estimates are plotted at the median date of the follow-up survey. Shading corresponds to the range of interview dates for alternating RCCS surveys.

Between 2015 and 2022, suppression among viremic people in the preceding survey increased by 1.31-fold (95% highest posterior density [HPD] 1.16–1.47, Supplementary Figure 10). In 2015, viral suppression was slightly less frequent among those with NNRTI and NRTI resistance in the previous survey (RR vs. those without resistance: 0.72, 95% HPD: 0.44–1.02 and 0.65, 95% HPD: 0.30–1.02, Figure 4). However, suppression among those with NNRTI or NRTI resistance increased significantly over the analysis period and by 2022 was comparable to those without resistance (RR: 1.15, 95% HPD: 0.93–1.39, and 1.14, 95% HPD: 0.86–1.42). This trend was observed throughout the study, suggestive of improved clinical management during the expansion of HIV treatment programs and potentially increased suppression due to DTG in the 2022 survey.

**Figure 5.**
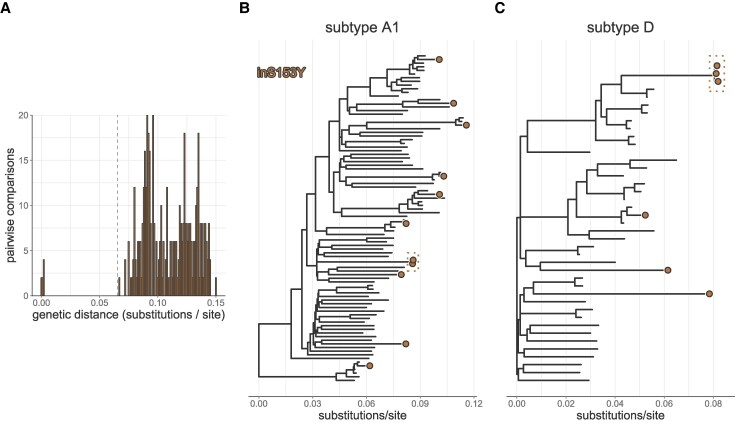
Genetic clustering of Rakai residents with viremic HIV with the inS153Y mutation. *A*, Pairwise viral genetic distance between Rakai residents with the inS153Y mutation, all of which were sampled after 8 February 2021. Dashed vertical line indicates the 99th percentile of pairwise genetic distance between people with HIV (PWH) without the inS153Y mutation sampled between 8 February 2021 and the end of the study period, 7 September 2023. *B*, Phylogenetic tree of subtype A1 HIV sequences from PWH with the inS153Y mutation (circles) and the 10 most closely related Rakai HIV sequences for each. *C*, Phylogenetic tree of subtype D HIV sequences from PWH with the inS153Y mutation (circles) and the 10 most closely related Rakai HIV sequences for each. In (*B*) and (*C*) clustered tips as identified in (*A*) are highlighted with dotted line.

To assess the transmission dynamics giving rise to the emergence of inS153Y during DTG scale-up, we integrated 2985 RCCS deep-sequenced samples with complete polymerase sequences with 274 from other participants in ongoing Rakai Health Sciences Program research studies (Methods, Supplementary Table 23). inS153Y was observed in 23/311 (7.4%) of deep-sequenced samples collected during or after the 2022 survey from 306 unique individuals (7.5%). Of these, 11 were with HIV subtype A1, 6 with subtype D, and 6 with recombinant subtypes. The mutation was universally observed as a minor variant, present in 5%–10% of sequencing reads and there was no clear pattern of it co-occurring with other resistance mutations (Supplementary Figures 11–13). Five individuals with inS153Y (21.74%) were linked to another individual with inS153Y at a genetic distance <0.065 substitutions/site, the 99th percentile of pairwise distances between viremic PWH without inS153Y sampled over the same epoch (Figure 5). We observed 2 inS153Y genetic clusters, one of size 3 (subtype D, 2 females and a male) and one of size 2 (subtype A1, female and a male), indicative of multiple instances of linkage through recent transmission.

## DISCUSSION

Here, we quantified HIV treatment utilization, viral load suppression, and ART resistance throughout a period of intensive scale-up of treatment and prevention efforts, including the transition to DTG-based first-line regimens. Viral suppression significantly increased over the study, reaching 90% of PWH by 2022. Resistance among txPWH fell by almost 50% due in part to increased suppression among those individuals with resistance. Despite no longer being a first-line regimen component, NNRTI resistance continued to increase in this setting. Intermediate/high-level DTG resistance was observed in only 2 treatment-experienced individuals with prior NRTI resistance. Concerningly, we observed the emergence and potential transmission of the inS153Y mutation among both txPWH and ptPWH following the scale-up of DTG use. These results provide critical insights into the dynamics of HIV treatment and suppression in an East-African setting with high-HIV burden with important implications for efforts to support reaching milestones such as the UNAIDS 95–95-95 targets.

This work corroborates findings on the impact of DTG on viral suppression among care-seeking PWH with population-based data. In South Africa, for example, DTG initiators were slightly more likely to achieve suppression after 12-months (83% vs. 81%) [6]. Among PWH on treatment, those who transitioned to DTG regimens were more likely to maintain suppression (94.3% vs. 82.1%) in the AFRICOS (Kenya, Nigeria, Tanzania, and Uganda) cohort [8] and overall suppression rate among DTG initiators was marginally, yet significantly, higher in South Africa (90.5% vs. 89.7%) [6]. We further show that population-scale viral load suppression among PWH increased significantly following DTG scale-up. This continues a trend ongoing since 2014 and likely reflects the combined impact of DTG and programmatic improvements in treatment-initiation and adherence.

Consistent with prior work [9, 29], we observed limited evidence of intermediate/high-level DTG resistance. Observed DTG resistance was associated with pre-existing NRTI resistance, augmenting evidence that effective DTG monotherapy can lead to treatment failure [5, 29]. However, inS153Y was observed among txPWH and ptPWH in 2022, despite not being observed earlier, with evidence suggesting transmission of this mutation. This pattern could be indicative of recent transmission from unsampled individuals with virological failure on DTG followed by reversion of deleterious mutations conferring high-level resistance. Observing inS153Y in this setting soon after the DTG transition highlights the potential for DTG resistance mutations to be selected for and transmitted in a high-burden setting and the value of population-based surveillance in rapidly identifying this phenomenon. Further, the continued increase in the prevalence of rtE138A (RPV resistance) among ptPWH requires consideration in light of efforts to roll-out long-acting injectable CAB/RPV as this mutation is associated with CAB/RPV failure [30]. Pre-treatment resistance testing, not currently routine in sub-Saharan Africa, may be useful to ensure individuals with rtE138A achieve durable suppression on CAB/RPV.

NNRTI and NRTI resistance among viremic txPWH declined throughout the study period and accelerated during DTG uptake. This is suggestive of increased suppression among those with resistance, as indicated by our analyses and reductions in acquired resistance. In 2022, nearly all (97.3%) of viremic txPWH were susceptible to DTG and the majority (72.1%) susceptible to all ART. As NRTI resistance reverts rapidly during treatment interruption [31], this suggests 2 cohorts of viremic txPWH: those with NRTI resistance who are transiently engaged in care and potentially at risk of DTG failure and those without resistance who are likely to be disengaged from care entirely. Those disengaged from care are exactly the people missed in clinic-based studies, reinforcing the utility of population-based surveillance to accurately quantify virological trends among all PWH, not just those engaged in care. As about half of viremic people are treatment-experienced, re-engaging this population in care is critical in reducing the population prevalence of viremia.

This study has limitations. We assessed DTG utilization among regional clinics, yet lacked data on individual-level regimens and could not estimate a direct effect of DTG on suppression. Drug presence assays and phenotypic resistance testing are not routinely conducted in the RCCS and we were unable to disentangle the role of adherence and resistance in viremia among txPWH. We also relied on self-reported treatment status, possibly leading to misclassification particularly among ptPWH. However, we suspect misclassification to be minimal given the divergent resistance motifs among txPWH and ptPWH as minimal (11%) ART use among pre-treatment RCCS participants was observed in previous work [32]. Finally, given the recent scale-up of DTG use in this setting, we may expect resistance to increase as time on DTG, and opportunities for emergence of DRMs, increases.

These results provide an encouraging view of the east-African HIV epidemic. At the end of follow-up, the most significant barriers to widespread suppression appear to be delays in treatment initiation and failure to achieve persistent adherence. The risk of DTG resistance appears minimal, particularly among ptPWH. The rapid emergence of inS153Y, however, suggests the need for ongoing viral genomic surveillance to maintain long-term efficacy. Broadly, our findings emphasize that ART resistance has not, to date, eroded the population-scale impacts of investments in developing novel ART-regimens and making them widely available. Critically, HIV care is suppressive, and not curative, and reductions in access to treatment threaten to rapidly erode many of these positive impacts [33–35]. As intermittent adherence to INSTI-based regimens is associated with a risk of acquired resistance [36], treatment interruptions may lead to wide-scale emergence of resistance against a highly effective regimen, thwarting major research investments. This would have devastating consequences for the health of people living in high-HIV burden communities as well as people globally given the international nature of HIV transmission [37].

## Supplementary Material

ciag161_Supplementary_Data

## Supplementary Data

Supplementary materials are available at *Clinical Infectious Diseases* online. Consisting of data provided by the authors to benefit the reader, the posted materials are not copyedited and are the sole responsibility of the authors, so questions or comments should be addressed to the corresponding author.

## References

[1] Trickey A, Sabin CA, Burkholder G, et al Life expectancy after 2015 of adults with HIV on long-term antiretroviral therapy in Europe and North America: a collaborative analysis of cohort studies. Lancet HIV 2023:e295–307. 36958365 10.1016/S2352-3018(23)00028-0 PMC10288029

[2] Cohen MS, Chen YQ, McCauley M, et al Antiretroviral therapy for the prevention of HIV-1 transmission. N Engl J Med 2016; 375:830–9. 27424812 10.1056/NEJMoa1600693 PMC5049503

[3] Gupta RK, Gregson J, Parkin N, et al HIV-1 drug resistance before initiation or re-initiation of first-line antiretroviral therapy in low-income and middle-income countries: a systematic review and meta-regression analysis. Lancet Infect Dis 2018; 18:346–55. 29198909 10.1016/S1473-3099(17)30702-8 PMC5835664

[4] Brown JA, Nsakala BL, Mokhele K, et al Viral suppression after transition from nonnucleoside reverse transcriptase inhibitor- to dolutegravir-based antiretroviral therapy: a prospective cohort study in Lesotho (DO-REAL study). HIV Med 2022; 23:287–93. 34632682 10.1111/hiv.13189 PMC9293184

[5] Schramm B, Temfack E, Descamps D, et al Viral suppression and HIV-1 drug resistance 1 year after pragmatic transitioning to dolutegravir first-line therapy in Malawi: a prospective cohort study. Lancet HIV 2022; 9:e544–53. 35905753 10.1016/S2352-3018(22)00136-9

[6] Dorward J, Sookrajh Y, Khubone T, et al Implementation and outcomes of dolutegravir-based first-line antiretroviral therapy for people with HIV in South Africa: a retrospective cohort study. Lancet HIV 2023; 10:e284–94. 37001536 10.1016/S2352-3018(23)00047-4 PMC10288006

[7] Kamori D, Barabona G, Maokola W, et al HIV viral suppression in the era of dolutegravir use: findings from a national survey in Tanzania. PLoS One 2024; 19:e0307003. 39141647 10.1371/journal.pone.0307003 PMC11324124

[8] Allahna E, Nicole D, Neha S, et al Virologic impact of the dolutegravir transition: prospective results from the multinational African cohort study. J Acquir Immune Deﬁc Syndr 2022; 91:285–9. 35980350 10.1097/QAI.0000000000003065 PMC9561234

[9] HIV drug resistance: brief report 2024. Geneva: World Health Organization.

[10] Pena MJ, Chueca N, D’Avolio A, Zarzalejos JM, Garcia F. Virological failure in HIV to triple therapy with dolutegravir-based firstline treatment: rare but possible. Open Forum Infect Dis 2019; 6:ofy332. 30631792 10.1093/ofid/ofy332 PMC6324549

[11] Huik K, Hill S, George J, et al High-level dolutegravir resistance can emerge rapidly from few variants and spread by recombination: implications for integrase strand transfer inhibitor salvage therapy. AIDS 2022; 36:1835–40. 35848510 10.1097/QAD.0000000000003288 PMC9594130

[12] Zhang WW, Cheung PK, Oliveira N, Robbins MA, Harrigan PR, Shahid A. Accumulation of multiple mutations in vivo confers cross-resistance to new and existing integrase inhibitors. J Infect Dis 2018; 218:1773–6. 30010985 10.1093/infdis/jiy428

[13] George JM, Kuriakose SS, Dee N, et al Rapid development of high-level resistance to dolutegravir with emergence of T97A mutation in 2 treatment-experienced individuals with baseline partial sensitivity to dolutegravir. Open Forum Infect Dis 2018; 5:ofy221. 30568974 10.1093/ofid/ofy221 PMC6172925

[14] Martin MA, Reynolds SJ, Foley BT, et al HIV drug resistance during antiretroviral therapy scale-up in Uganda, 2012–19: a population-based, longitudinal study. Lancet Microbe 2025; 6:101218. 41319670 10.1016/j.lanmic.2025.101218 PMC12722182

[15] Chang LW, Grabowski MK, Ssekubugu R, et al Heterogeneity of the HIV epidemic in agrarian, trading, and fishing communities in Rakai, Uganda: an observational epidemiological study. Lancet HIV 2016; 3:e388–96. 27470029 10.1016/S2352-3018(16)30034-0 PMC4973864

[16] Gall A, Ferns B, Morris C, et al Universal amplification, next-generation sequencing, and assembly of HIV-1 genomes. J Clin Microbiol 2012; 50:3838–44. 22993180 10.1128/JCM.01516-12 PMC3502977

[17] Bonsall D, Golubchik T, de Cesare M, et al A comprehensive genomics solution for HIV surveillance and clinical monitoring in low-income settings. J Clin Microbiol 2020; 58:e00382–20. 32669382 10.1128/JCM.00382-20 PMC7512176

[18] Fogel JM, Bonsall D, Cummings V, et al Performance of a high-throughput next-generation sequencing method for analysis of HIV drug resistance and viral load. J Antimicrob Chemother 2020; 75:3510–6. 32772080 10.1093/jac/dkaa352 PMC7662169

[19] Shafer RW . Rationale and uses of a public HIV drug-resistance database. J Infect Dis 2006; 194:S51–8. 16921473 10.1086/505356 PMC2614864

[20] Rhee S-Y . Human immunodeficiency virus reverse transcriptase and protease sequence database. Nucleic Acids Res 2003; 31:298–303. 12520007 10.1093/nar/gkg100 PMC165547

[21] Zou G . A modified poisson regression approach to prospective studies with binary data. Am J Epidemiol 2004; 159:702–6. 15033648 10.1093/aje/kwh090

[22] Margot NA, Ram RR, White KL, Abram ME, Callebaut C. Antiviral activity of HIV-1 integrase strand-transfer inhibitors against mutants with integrase resistance-associated mutations and their frequency in treatment-naïve individuals. J Med Virol 2019; 91:2188–94. 31389026 10.1002/jmv.25564

[23] The Montreal Primary HIV (PHI) Cohort Study Group; Oliveira M, Ibanescu R-I, et al Selective resistance profiles emerging in patient-derived clinical isolates with cabotegravir, bictegravir, dolutegravir, and elvitegravir. Retrovirology 2018; 15:56. 30119633 10.1186/s12977-018-0440-3 PMC6098636

[24] McCluskey SM, Kamelian K, Musinguzi N, et al Pre-treatment integrase inhibitor resistance is uncommon in antiretroviral therapy-naive individuals with HIV-1 subtype A1 and D infections in Uganda. AIDS 2021; 35:1083–9. 33635845 10.1097/QAD.0000000000002854 PMC8102316

[25] Rhee S-Y, Grant PM, Tzou PL, et al A systematic review of the genetic mechanisms of dolutegravir resistance. J Antimicrob Chemother 2019; 74:3135–49. 31280314 10.1093/jac/dkz256 PMC6798839

[26] Bacheler L, Jeffrey S, Hanna G, et al Genotypic correlates of phenotypic resistance to efavirenz in virus isolates from patients failing nonnucleoside reverse transcriptase inhibitor therapy. J Virol 2001; 75:4999–5008. 11333879 10.1128/JVI.75.11.4999-5008.2001 PMC114903

[27] Tisdale M, Kemp SD, Parry NR, Larder BA. Rapid in vitro selection of human immunodeficiency virus type 1 resistant to 3′-thiacytidine inhibitors due to a mutation in the YMDD region of reverse transcriptase. Proc Natl Acad Sci U S A 1993; 90:5653–6. 7685907 10.1073/pnas.90.12.5653 PMC46779

[28] Brun-Vézinet F, Descamps D, Ruffault A, et al Clinically relevant interpretation of genotype for resistance to Abacavir. AIDS 2003; 17:1795–802. 12891065 10.1097/00002030-200308150-00008

[29] Loosli T, Hossmann S, Ingle SM, et al HIV-1 drug resistance in people on dolutegravir-based antiretroviral therapy: a collaborative cohort analysis. Lancet HIV 2023; 10:e733–41. 37832567 10.1016/S2352-3018(23)00228-X PMC10913014

[30] Kityo C, Mambule IK, Sokhela SM, et al Randomized trial of Cabotegravir and Rilpivirine Long-acting in Africa (CARES):Week 96 Result. In: Randomized Trial of Long-Acting Cabotegravir and Rilpivirine in Africa (CARES): Week 96 Results. San Francisco, CA: 2025. Available at: https://www.croiconference.org/abstract/3613-2025/.

[31] Paquet AC, Baxter J, Weidler J, et al Differences in reversion of resistance mutations to wild-type under structured treatment interruption and related increase in replication capacity. PLoS One 2011; 6:e14638. 21297946 10.1371/journal.pone.0014638 PMC3031504

[32] Grabowski MK, Reynolds SJ, Kagaayi J, et al The validity of self-reported antiretroviral use in persons living with HIV: a population-based study. AIDS 2018; 32:363–9. 29194115 10.1097/QAD.0000000000001706 PMC6171354

[33] Ratevosian J, Millett G, Honermann B, et al PEPFAR under review: what's at stake for PEPFAR's future. The Lancet 2025; 405:603–5. 10.1016/S0140-6736(25)00258-2 39929219

[34] Tram KH, Ratevosian J, Beyrer C. By executive order: the likely deadly consequences associated with a 90-day pause in PEPFAR funding. J Int AIDS Soc 2025; 28:e26431. 39996580 10.1002/jia2.26431 PMC11851316

[35] Forsythe SS, McGreevey W, Whiteside A, et al Twenty years of antiretroviral therapy for people living with HIV: global costs, health achievements, economic benefits. Health Aff (Millwood) 2019; 38:1163–72. 31260344 10.1377/hlthaff.2018.05391

[36] Lepik KJ, Harrigan PR, Yip B, et al Emergent drug resistance with integrase strand transfer inhibitor-based regimens. AIDS 2017; 31:1425–34. 28375875 10.1097/QAD.0000000000001494

[37] Wertheim JO, Oster AM, Hernandez AL, Saduvala N, Bañez Ocfemia MC, Hall HI. The international dimension of the U.S. HIV transmission network and onward transmission of HIV recently imported into the United States. AIDS Res Hum Retroviruses 2016; 32:1046–53. 27105549 10.1089/aid.2015.0272 PMC5067842

